# An Assessment Framework for the Training of General Practitioners and Specialists Based on EPAs

**DOI:** 10.3389/fpubh.2022.896097

**Published:** 2022-07-07

**Authors:** Shenshen Gao, Na Li, Xinqiong Wang, Yi Yu, Ren Zhao, Virgínia Trigo, Nelson Campos Ramalho

**Affiliations:** ^1^Departments of Technology Development and Pediatric, Ruijin Hospital, Shanghai Jiao Tong University School of Medicine, Shanghai, China; ^2^Clinical Research and Development Center of Shanghai Municipal Hospitals, Shanghai Shenkang Hospital Development Center, Shanghai, China; ^3^Department of Human Resources Management and Organizational Behavior, ISCTE Instituto Universitário de Lisboa, Av.das Forcas Armadas, Lisboa, Portugal; ^4^Department of Tropical Diseases, Key Laboratory of Tropical Translational Medicine of Ministry of Education, NHC Key Laboratory of Tropical Disease Control, The Second Affiliated Hospital of Hainan Medical University, School of Tropical Medicine, Hainan Medical University, Haikou, China; ^5^Department of General Surgery, Ruijin Hospital, Shanghai Jiao Tong University School of Medicine, Shanghai, China; ^6^School of Health Services Management, Southern Medical University, Guangzhou, China

**Keywords:** pediatricians, medical service, training, entrustable professional activities, structural model

## Abstract

**Purpose:**

The purpose of this study is to explore the practicality and feasibility of EPA (level 4 theory) for establishing medical training and service model in China.

**Method:**

We opted for a mixed qualitative and quantitative research method, considering both explanatory and exploratory sequential designs. The qualitative research comprehended focus groups and interviews conducted with two panels of experts. The quantitative research was conducted to collect data about the applicability of international entrustable professional activities (EPAs) pediatrics standards in the Chinese context by organizing a seminar with a sample of 60 pediatricians. A questionnaire was designed with EPAs and distributed within professional networks. Structural equation modeling and statistical analysis were used to process the data.

**Results:**

In this study, Medical Service-Groups Model (MSGM) with four levels was successfully established to measure the correlation between specialized and general EPAs. As expected, results showed that specialized EPAs were built on top of general EPAs. There may be a mediating mechanism that general EPAs contribute to the lower level of specialization EPAs. In addition, levels 1 and 2 were primarily needed to lay the groundwork for levels 3 and 4, and these higher levels of EPAs were still the most informative for specialized Gastroenterology EPAs.

**Conclusions:**

The diagnosis and treatment level of primary general practitioners, as the basis of the pediatric medical service chain, affected the clinical disposal ability of specialists. The establishment of MSGM provided a theoretical basis for the linkage training of general practitioners and specialist physicians. In future studies, scholars must explore China's EPAs based on unique national conditions.

## Introduction

Medical development has always been a worldwide subject of research. The World Health Organization (WHO) introduced a basic framework for an integrated health service system in 2016 and made it a global health strategy contributing to sustainable development goals. Despite the rapid development of medical care in China in recent decades, there are still many problems such as the scarcity of medical resources, the imbalance between supply and demand, and the imperfect primary medical system (1, 2). There are many factors contributing to this effect such as a big workload for specialist physicians, an insufficient supply of general physicians, and patients' preferences for community hospitals (3).

There are about 279 million children under the age of 18 years in China, accounting for 15% of children worldwide (4). Therefore, pediatric health care assumes enormous national responsibility, and pediatricians play an important role in China's health care system and constitute a core component of pediatric care resources. The crisis facing pediatric medicine in China, namely the burnout of pediatricians that may place both physicians and patients at risk, (5, 6) has received considerable publicity recently (7). In this context, it is of great significance to integrate regional pediatric medical resources, strengthen the support of primary pediatric medical institutions, improve the ability of pediatric medical services, optimize the allocation of pediatric medical resources, and improve medical efficiency and satisfaction.

Within the profession, the specialty of pediatric gastroenterology is rapidly evolving and increasingly recognized and accepted worldwide (8). The unique characteristics of pediatric gastroenterologists are that they have the potential to be experts in much of the anatomy and physiology of the human body (9). Trainees must have the capacity to analyze and integrate the clinical data, rather than limiting their thought processes to a specific organ or part of the gastrointestinal tract. In addition, the practitioners require routine consulting and collaborating with myriad allied providers and many of the diseases encountered are also related to other subspecialties, necessitating cooperative relationships with these experts. Therefore, the field of pediatric gastroenterology is multidisciplinary and a representative clinical discipline.

To alleviate the contradiction between the increasing demand for medical treatment and the scarcity of pediatrician resources and to better play the radiation effect of medical resources in community hospitals, this study introduced the concept of entrustable professional activities (EPAs) as a tool to assess the competence of pediatric gastroenterologists. EPAs were often used to observe and measure competencies (10, 11). Various courses have been described for suitable EPAs, which involved local or national expert groups (12–14). EPAs are defined as a comprehensive set of responsibilities that physicians (specialists or general practitioners) should be able to perform in their particular field. They must be detailed enough to set the expectations of trainees and guide the assessment and entrustment decisions of supervisors (15). EPAs also provide curriculum developers with tangible goals to align training with clinical practice (16).

In this research, EPAs theory was employed to explore a training model for Chinese pediatricians *via* qualitative and quantitative analysis. The model can be used for the further education of Chinese pediatricians and pediatric skill training for general physicians in communities or the Co-training of general pediatricians and specialist pediatricians. We studied the Co-ordination possibilities between general pediatricians and specialist pediatricians in terms of entrustable professional activities, which served as the prelude for the prospective research of the functional construction of the referral medical system between community, central, and teaching hospitals. The Co-ordination between general and specialist pediatricians can help establish a sound treatment order.

## Method

### Research Design

We opted for a mixed-methods approach as it offers the possibility of joining the strengths of both qualitative and quantitative (17) methods. In designing this mixed-methods study, we considered both explanatory and exploratory sequential designs (18). As our major intention was exploratory in nature, that is, we were motivated first to uncover freely generated interpretations of EPA in pediatrics in China, we deployed the exploratory sequential design from the perspective of an inductive approach. With the insight it provided from qualitative data analysis, we could design a questionnaire that allowed for a comprehensive data collection, thus integrating all variables into a single explanatory model.

### Qualitative Research

The focus group and expert interviews were conducted with a semi-structured interview technique. The two kinds of interviews were semi-guided and adopted the same questions, although they could be changed to better capture what the interviewees might be concerned about. This study was organized in a total of two focus group interviews and four expert interviews: all the participants in the first interview are pediatricians (specialists and ordinary pediatricians). The participants in the second interview are nurses, medical technicians, scientific research personnel, management personnel, and social experts.

### Quantitative Research

We used the EPAs items that have been widely certified and used to explain, verify, and amend a model through the development of practice activities (sourced from EPAs) among Chinese pediatricians.

#### Examining Whether the EPAs Are Consistent With Chinese Pediatricians

The 17 items (Community Pediatricians) of EPAs Theory formulated by the American Academy of Pediatrics (AAP) and the five items (Practice Activities for Pediatric Digestion Specialty) drafted by the managerial level of the North American Society for Pediatric Gastroenterology, Hepatology, and Nutrition and AAP were chosen as a reference for making the questionnaire for investigation and survey. The top priority was to examine where the EPAs were consistent with the daily medical practice activities of Chinese pediatricians and pediatric specialists on digestion.

The researchers promoted a seminar in the form of an enlarged session during an executive meeting of the Shanghai Central Pediatrician Medical Treatment Partnerships with over 60 participants, including authoritative experts at home and abroad, daily clinical teachers who are experienced in both teaching and clinical work, and who hold intermediate professional titles or above. The authors presided over the seminar and briefed on the research background and problems that needed to be solved, introducing the research progress of EPAs and relevant theories. At the meeting, 22 EPAs (covering pediatric general practitioners and specialists on digestion) were given out to the experts to judge whether the items were in accord with the daily practice activities.

To enable these experts to better understand the meaning of practice activities, the full name and specific functions of each practice activity were listed. For the convenience of follow-up classification, the materials distributed to senior professional title experts were color-printed while the materials to physicians with intermediate or lower professional titles were printed in white and black. Each item of the materials issued at the seminar was attached with specific activity notes. After the seminar, supplements and corrections were made based on the feedback of experts.

#### Questionnaire Design and Measures

The questionnaire (19) covered relevant demographic information, occupational information, types of hospitals, and professional characteristics. The 5-point Likert scale was used to measure specific practice activities of 22 EPAs (pediatricians plus pediatric specialists on digestion).

Entrustable professional activities for general pediatrics were measured using 17 EPAs as found in the qualitative and the first quantitative phase. Participants were requested to answer on a 5-point Likert scale ranging from 1 (not important at all) to five (extremely important) to which extent they agreed that the described EPA should be performed frequently by pediatric gastroenterologists in community hospitals.

Entrustable professional activities for gastroenterological pediatricians were measured with five EPAs as found in the qualitative and the first quantitative phase. Participants were requested to answer on a 5-point Likert scale ranging from 1 (not important at all) to 5 (extremely important) to which extent they agreed that the described specialized EPA should be performed frequently by pediatric gastroenterologists in community hospitals.

Considering the professionalism of the contents in the questionnaires, the eligible respondents should be pediatricians, pediatric specialists on digestion, and other groups closely related to pediatrics. Therefore, the questionnaire was sensibly distributed to the groups in this industry by using special channels. To ensure the quality of the sample collected, the questionnaires were handed to members of China's pediatric academic organizations and participants in the academic meetings.

Finally, with guaranteed validity and reliability, we will show the test results: (a) if the EPA levels of complexity operate in parallel or in a hierarchical way, (b) which EPA levels predict each specialized EPAs, and (c) which individual EPAs predict each specialized EPAs, so to uncover the competencies that leverage EPAs. In this way, we intend to understand if the EPAs apply to pediatrics in China. The final model will offer a structural view for the linkage training of general practitioners and specialists.

### Statistical Analysis

We confirmed data suitability using the Kaiser–Meyer–Olkin (KMO) index of sampling adequacy, adopted Covariance Base SEM (CB-SEM) and Partial Lease Square SEM (PLS-SEM) Structural Equations Modeling, and tested with PROCESS macro available in SPSS (20) and PLS-SEM software (Warp-PLS) (21). Statistical analyses were performed using IBM SPSS Statistics 20 and AMOS 17.0. For all tests, the statistical significance level was set at *P* < 0.05.

## Results

### Data Description of the Sample

The sample comprises 776 valid answers originating from almost all the Chinese provinces with about two-thirds of the participants being from Hebei, Shanghai, Hubei, Jiangsu, Hainan, Hainan, Liaoning, and Guangdong (Supplementary Figure 1 and Supplementary Table 1).

The type of pediatrician crossed with the type or nature of hospital is depicted in the following tables (Supplementary Tables 2, 3). It is clear that specialists are mostly working in community hospitals and that the largest sample comes from such hospitals.

### Reflective vs. Formative Nature of EPAs

The opinions of experts holding a title of a senior professional post were mainly taken into consideration. Among the reviewed materials, research on the suggestions of doctors holding a title of a senior professional post was conducted. It was found that all 22 EPAs (pediatric general practitioners plus pediatric specialists on digestion), through expert review, were basically in line with the practice activities of Chinese pediatricians.

Validity analysis was used to know if the items under study were reasonably and meaningfully treated as reflecting a latent construct (22). Validity can be approached using a factor analysis where KMO value, commonalities, explained variance, and factor-loading Co-efficients are considered to judge the suitability of the analysis. KMO serves to identify if the data intercorrelation is high enough to allow the factor analysis. Commonalities should attain at least the 0.5 threshold. When a given item does not do this, it does not converge with the other items in the factor analysis and should be excluded.

The variance explanation rate represents the information extraction quality and should be at least 60. Finally, factor loadings measure the magnitude in which a given item value reflects the corresponding latent construct.

The factor analysis validity indicators suggested that there was enough high level of intercorrelations to allow it (KMO = 0.993). However, there were many cases of insufficient commonalities (Supplementary Table 4) and the explained variance was below the comfortable level (*R*^2^ = 48.5%).

By sequentially removing items that showed lower commonality, we were able to obtain an efficient two-factor solution that had good KMO (.829) with commonality above.500 for all items, despite explaining only 58.5% of the total variance after rotation (Varimax). The remaining items corresponded to only seven EPAs, but one of them (EPAs #16) should be deleted based on cross-loading analysis. Therefore, the final factor rotation matrix contained only six EPAs (KMO = 0.771, commonalities all above.576, *R*^2^ = 60.9%) and had been organized in pediatricians' minds into a two-factor reflective structure (Supplementary Table 5).

This was relevant information, as it pertained to how extensively professionals integrated EPAs into a set of cognitive categories but is not workable for this research. These findings indicated that pediatricians had a common understanding of these six EPAs as organized around two major categories (data-based actions and intervention actions), but it leaves out very important EPAs. Because this research emphasizes performed activities, it was not a requirement that EPAs were organized around reflective constructs. Therefore, we concluded that EPAs should be considered as a formative structure. The reflective structure is the unobservable construct, which consists of the reflective indicators and the error term for each indicator. For formative structure, the items describe and define the construct rather than vice versa. The constructs comprised of these causal indicators along with a disturbance term. A paralleled scenario occurred for specialized gastrointestinal EPAs (SGEPAs) with the exploratory factor analysis showing a high level of intercorrelations (KMO = 0.824), except one case that had insufficient commonality (Supplementary Table 6) and explained variance below the comfort level (*R*^2^ = 56.1%).

Following the same rationale, we concluded that the construct for SGEPAs was in line with the previous choice for treating EPAs as formative. For such purposes, we will treat general EPAs and SGEPAs in the same way.

### Reliability and Validity Analyses for General and Specialized Gastrointestinal EPAs

Since validity is based on the formative nature of constructs, we need to verify if the measures are reliable. Reliability concerns the extent to which a given questionnaire measures the same construct. It was commonly expressed by Cronbach's alpha, which attained the value of 0.70 (22). Findings were shown in Supplementary Table 7.

As may be seen in the above table, the reliability Co-efficients of General EPAs and SGEPAs were 0.883 and 0.801, respectively. Regarding the item deleted, α Co-efficient, the reliability Co-efficient of both cases has no significant improvement. As for CITC, there were many cases where the value fell below 0.60, indicating problems. In conclusion, the overall scale is reliable, but there are problems with specific items, probably because of its formative nature.

### Testing the Hierarchical Model of EPAs

We speculated that general EPAs followed some sort of sequential structure, from simple to complex, from early to late, and from general to specialized. To uncover a meaningful structure, we reasoned that clinical learning processes took place in stages: (1) to gather all required information to reach a good diagnosis; (2) to understand macro-level dimensions that may impact the accuracy of the diagnosis; (3) to be capable of bridging with other services; and finally, (4) to deliver a best practice-based treatment while being able to lead clinically.

The first phase: when faced with any clinical situation, pediatricians need to properly use screening tools to gather information. This information will be more accurate when the physician can establish a positive relationship with the patient's family as well as colleagues. A family that is trusting and willing to Co-operate will disclose more information. Likewise, colleagues with whom one can have an open communication channel will also contribute to triangulating and clarifying doubtful situations.

The second phase: when faced with an emergency, pediatricians must be able to consider macro information related to risk groups, epidemiology, and understand services for referring emergency cases to pediatricians.

The third phase: many cases will require the intervention of other medical area professionals. These cases require a sense of importance and know how to transfer to other services to provide for clinical needs that may fall outside the scope of pediatricians.

The fourth phase: delivering best practice service implies knowing and observing referral guidelines while keeping a focus not only on treatment but also on the need to lead oneself into learning more while leading others. This level is the most complex as it comprehends practices that are usually allocated only to pediatricians in community hospitals.

Because such logic might not resonate with all pediatricians, we have subjected this proposal to the validation of expert pediatricians. We elected three senior-level experts and three more experts but with the medium position. The results may be found in Supplementary Table 8.

The interpretation allows the attribution of the following objectives linked to each EPA level:

1st level: establishing conditions to gather information for diagnosis
2nd level: first diagnosis and treatment
3rd level: bridging/referring to other services
4th level: observing best practices and leading

The four levels, named as Medical Service-Groups Model (MSGM), had an expected contribution to the overall job performance of a pediatrician while simultaneously being linked in a sequence from the simplest to the more complex. This configured a sequential mediation model with three paths. The sequential mediation model is single-input and single-output, with one path leading to the end. There is an only adjacent relationship between layers and no cross-layer connection. The first path linked to level 1 with level 2, the second path linked to level 2 with level 3, and the third path linked to level 3 with level 4. We believed that it is also reasonable to expect relations between these levels that bypass the sequential mediation making it partial. We assumed that there is a partial mediation between levels 1 and 4, which occurs through a sequential positive relationship between levels 2 and 3.

Based on this rationale, we hypothesized that:

Hypothesis 1: Level 2 mediated the positive relationship between Levels 1 and 3 $\left(1\overset{2}{↔}3\right)$
Hypothesis 2: Level 3 mediated the positive relationship between Levels 1 and 4$\left(1\overset{3}{↔}4\right)$
Hypothesis 3: There was a sequential mediation by Level 2 and Level 3 in the positive relationship between Levels 1 and 4 $\left(1\underset{3}{\overset{2}{↔}}4\right)$.

#### Hierarchical EPAs Structure of MSGM With CB Models

The MSGM was depicted in Supplementary Figure 2, where X was Level 1, Y was Level 4, and M1 and M2 were Levels 2 and 3, respectively. The results showed that there were six direct effects and three indirect effects between X and Y operating simultaneously.

The relation between Levels 1 and 2 had considerable power that the explained variance was 37.1% corresponding to a significant F (1,774) value of 458.1993 (*P* < 0.01). The direct effect of Level 1 on Level 2 had a Co-efficient of.659.

The joint relation of Levels 1 and 2 on Level 3 also had considerable, with an explained variance of 49.4% corresponding to a significant F (2,773) value of 377.6766 (*P* < 0.01). The direct effect of Levels 1 and 2 was also significant, with a magnitude of.301 and.423, respectively.

The join relation of all preceding levels on Level 4 was slightly stronger than the previous with an explained variance of 50% corresponding to a significant F (3,772) value of 258.2555 (*P* < 0.01). The direct effects of Levels 1, 2, and 3 were all significant with a magnitude of 0.102, 0.180, and 0.342, respectively.

The totally standardized indirect effect of Level 1 on Level 3 through Level 2 was significant with a magnitude of 0.1508. This supported Hypothesis 1$\left(1\overset{2}{↔}3\right)$.

The totally standardized indirect effect of Level 1 on Level 4 through Level 3 was significant with a magnitude of 0.1312. This supported Hypothesis 2$\left(1\overset{3}{↔}4\right)$.

The totally standardized indirect effect of Level 1 on Level 4 through both Levels 2 and 3 was significant with a magnitude of 0.1215. This supported Hypothesis 3$\left(1\underset{3}{\overset{2}{↔}}4\right)$.

Supplementary Table 9 summarized the findings from mediation testing and its respective classification. The results pertaining to mediation were shown in Supplementary Figure 3.

#### Hierarchical EPAs Structure of MSGM With PLS Models

The Average path Co-efficient (APC) (23) should be statistically significant (*P* < 0.05) and expressed the average association Co-efficients for direct effects established between latent variables. The Average R-squared (ARS) (24) and Average adjusted R-squared (AARS) should also be statistically significant, and the difference between the two should be no more than 5%. Other sets of indices are multicollinearity. The software offers calculations on the Average block VIF (AVIF) (25) and Average full collinearity VIF (AFVIF) that ideally should fall below 3.3 (values up to 5 are also within the acceptance range). These indicators are particularly important as they can show if common method bias occurred (26). Another important indicator is the Tenenhaus GoF (GoF) (27) which is expressed as being small (0.01≤ GOF <0.25), medium (0.25≤ GOF <0.36), or large (GOF over 0.36) and measures the explanatory power of the model. Other issues that may hamper the quality of a PLS-SEM model concern specific patterns of association between the values of two variables in such a way that false negatives may emerge. Sympson's paradox ratio (SPR) is useful to detect these. It should not fall below 0.70.

In addition, the R-Squared Contribution Ratio (RSCR) verifies if the model has any case of negative R-squared contribution, which would indicate the wrongly designed dependence direction of the hypotheses. Values over 0.9 indicate no problem with this issue. Also, statistical suppression is a problem that may occur when the absolute beta value is higher than the correlation between the two latent variables (28) and the software incorporates an index (SSR) that indicates the extent to which this might have occurred. Values of 0.70 or above indicate that statistical suppression did not occur. Finally, the non-linear bivariate causality direction ratio (NLBCDR) indicates how much beta Co-efficients between two latent variables may change when using non-linear algorithms and inverting the direction of causality. Perfect situations are indicated by a value of NLBCDR of 1, but the acceptable threshold is set to 0.70.

In the case of the current sequential mediation model, all values excluded validity and quality problems associated with the model. Correlation degree and explanatory variance were significant (APC = 0.369, ARS = 0.465, AARS = 0.464; *P* < 0.001). Likewise, there was no obvious multicollinearity problem (AVIF = 1.795, AFVIF = 2.101), and the model fitted the data well (GOF = 0.480). The SPR was 1 and Sympson's paradox was thus not a matter of concern, which was consistent with the SSR value of 1. In the direction of influence, the improvement was not observed in RSCR and NLBCDR (1 for both values) from reversing direction.

Variables also had high reliability. Composite Reliability (CR) reached threshold of 0.70 for all EPA levels (CR Level 1–4: 0.792, 0.782, 0.819, 0.814). The distributions were unimodal in both Rohatgi–Szekely and Klaassen–Mokveld–van ES tests, indicating that the results were reliable. Results showed that all direct paths were statistically significant (Supplementary Figure 4).

The indirect effects of the two-path and the three-path were significant. The relationship mediation between Levels 1 and 3 *via* Level 2 presented a significant value of 0.287 (*P* < 0.01). Likewise, the relationship mediation between Levels 1 and 4 *via* Level 3 presented a significant value of 0.310 (*P* < 0.01). Finally, the three-path mediation model through Levels 2 and 3 also presented a significant value of 120 (*P* < 0.01) (Supplementary Table 10).

These results also supported all three hypotheses. Comparatively, although the path Co-efficients (and consequently indirect effects) in the PLS-SEM model were substantially larger than those in the CB-Process, the statistical significance remained equivalent for all studied paths. This ensured that the existing paths were not affected by data analysis technique options.

### Specialized EPAs Dependence on General EPAs

Another issue of relevance to uncover the structure of EPAs was how they relate to the SGEPAs. Because specialized learning goals cannot be achieved at the expense of previously acquired learning and skills, we assume that:

Hypothesis 4: All EPA levels are positively associated with each of the SGEPAs.

We derived six sub-hypotheses from H4a to H4f, one per each. Furthermore, we speculated that the magnitude of association between general EPAs and SGEPAs differed in the sense of being stronger in the more complex general EPAs.

Therefore, we hypothesize that:

Hypothesis 5: There will be stronger associations between higher complexity EPA levels and SGEPAs than those found between the lower level and SGEPAs.

In this case, six sub-hypotheses were also derived from H5a to H5f. The CB-SEM method is used to analyze the covariance structure of variables. It mainly tests the applicability of theories and is suitable for testing theoretical models (validation), while the PLS-SEM method is used to analyze the principal component structure of variables. Mainly in the interpretation of variance (testing whether causality has a significant relationship), suitable for the construction of the theoretical model (exploratory), but also used to verify the causal relationship discussed. Because CB-SEM and PLS-SEM were previously used for analysis, we repeated this procedure for the new predictive models of SGEPAs, using multiple OLS regression with SPSS 24 and WarpPLS-SEM 6.0.

#### Specialized EPA Dependence on General EPA With CB Models

The results showed that each of the dependent regression analyses was separated. For GI EPA 1-5, the OLS multiple regression explained an adjusted variance of 36.9, 28.9, 28.0, 25.9, and 17.7%, respectively, where Levels 3 and 4 were significant predictors (Tables 1–5). This supported both Hypotheses 4 and 5.

**Table 1:** OLS regression for GI EPA 1^a^.

**Model**	**B** **(unstandardized)**	**Standard error**	**Beta** **(standardized)**	**t**	**Sig**.	**Collinearity statistics**	
						**Tolerance**	**VIF**
(Constant)	0.005	0.202		0.024	0.981		
EPA GI Lev1	0.003	0.055	0.002	0.060	0.952	0.551	1.815
EPA GI Lev 2	0.095	0.056	0.072	1.713	0.087	0.465	2.151
EPA GI Lev 3	0.340	0.064	0.231	5.293	0.000	0.428	2.337
EPA GI Lev 4	0.687	0.074	0.377	9.340	0.000	0.499	2.004

a *Dependent Variable: B18 (GI EPA 1). Care acute/chronic GI disease*.

**Table 2:** OLS regression for GI EPA 2^a^.

**Model**	**B** **(unstandardized)**	**Standard error**	**Beta** **(standardized)**	**t**	**Sig**.	**Collinearity statistics**	
						**Tolerance**	**VIF**
(Constant)	0.324	0.222		1.454	0.146		
EPA GI Lev1	–0.040	0.061	–0.027	–0.659	0.510	0.551	1.815
EPA GI Lev 2	0.096	0.061	0.070	1.567	0.117	0.465	2.151
EPA GI Lev 3	0.329	0.071	0.216	4.658	0.000	0.428	2.337
EPA GI Lev 4	0.638	0.081	0.338	7.880	0.000	0.499	2.004

a *Dependent Variable: B19 (GI EPA 2). Care acute/chronic hepatobiliary disease*.

**Table 3:** OLS regression for GI EPA 3^a^.

**Model**	**B** **(unstandardized)**	**Standard error**	**Beta** **(standardized)**	**t**	**Sig**.	**Collinearity statistics**	
						**Tolerance**	**VIF**
(Constant)	0.839	0.207		4.049	0.000	0.551	1.815
EPA GI Lev1	0.037	0.057	0.027	0.652	0.515	0.465	2.151
EPA GI Lev 2	–0.008	0.057	–0.007	–0.147	0.883	0.428	2.337
EPA GI Lev 3	0.328	0.066	0.232	4.983	0.000	0.499	2.004
EPA GI Lev 4	0.588	0.075	0.336	7.797	0.000		

a *Dependent Variable: B20 (GI EPA 3). Diagnose and manage common GI/hepatobiliary diseases*.

**Table 4:** OLS regression for GI EPA 4^a^.

**Model**	**B** **(unstandardized)**	**Standard error**	**Beta** **(standardized)**	**t**	**Sig**.	**Collinearity statistics**	
						**Tolerance**	**VIF**
(Constant)	0.391	0.223		1.755	0.080		
EPA GI Lev1	0.137	0.061	0.094	2.253	0.025	0.551	1.815
EPA GI Lev 2	0.186	0.061	0.138	3.033	0.003	0.465	2.151
EPA GI Lev 3	0.276	0.071	0.184	3.900	0.000	0.428	2.337
EPA GI Lev 4	0.348	0.081	0.188	4.288	0.000	0.499	2.004

a *Dependent Variable: B21 (GI EPA 4). Assess and provide counseling regarding nutrition*.

**Table 5:** OLS regression for GI EPA 5^a^.

**Model**	**B** **(unstandardized)**	**Standard error**	**Beta** **(standardized)**	**t**	**Sig**.	**Collinearity statistics**	
						**Tolerance**	**VIF**
(Constant)	0.881	0.259		3.406	0.001		
EPA GI Lev1	0.026	0.071	0.016	0.368	0.713	0.551	1.815
EPA GI Lev 2	0.036	0.071	0.024	0.500	0.617	0.465	2.151
EPA GI Lev 3	0.218	0.082	0.132	2.652	0.008	0.428	2.337
EPA GI Lev 4	0.609	0.094	0.299	6.471	0.000	0.499	2.004

a *Dependent Variable: B22 (GI EPA 5). Using endoscopy*.

Overall, hypothesis 4 was fully supported, thus suggesting that there was empirical evidence that SGEPAs were built on top of general EPAs. Likewise, Hypothesis 5 suggests that an established stronger association between higher general EPA levels (3 and 4) with SGEPAs was globally supported to the exception of GI EPA 4. Furthermore, there was no indication of multicollinearity, meaning that the explained variance was not exaggerated due to inter EPA correlations.

Interestingly, the dependence of SGEPAs was stronger in the simplest GI EPAs, suggesting a possible mediating mechanism by which general EPAs contributed directly or indirectly through the lower levels of SGEPAs.

#### Specialized EPA Dependence on General EPA With PLS-SEM Models

In the first model (General EPA levels and SGEPAs 1), APC = 0.290 (*P* < 0.001), ARS = 0.443 (*P* < 0.001), and AARS = 0.441 (*P* < 0.001) (Table 6), indicating that it matched the requirements of validity and quality.

**Table 6:** Association Co-efficients general EPAs-GI EPA 1 for PLS-SEM.

	**EPA_level 1**	**EPA_level 2**	**EPA_level 3**	**EPA_level 4**
EPA_level 1				
EPA_level 2	0.610^*^			
EPA_level 3	0.313^*^	0.470^*^		
EPA_level 4	0.107	0.295^*^	0.417^*^	
EPA_GI 1	0.011	0.056	0.242^*^	0.374^*^

*P < 0.001.

Multicollinearity was also ruled out (AVIF = 1.926, AFVIF = 2.064). The model fitted well (Tenenhaus GoF = 0.514), without Sympson's paradox problem (SPR = 1.000). SPR and SSR were both equal to 1, so there were no Simpson paradoxes problems due to data distortion. And both RSCR and NLBCDR were equal to 1, indicating that the reversal direction had not been improved.

**Figure 1:**
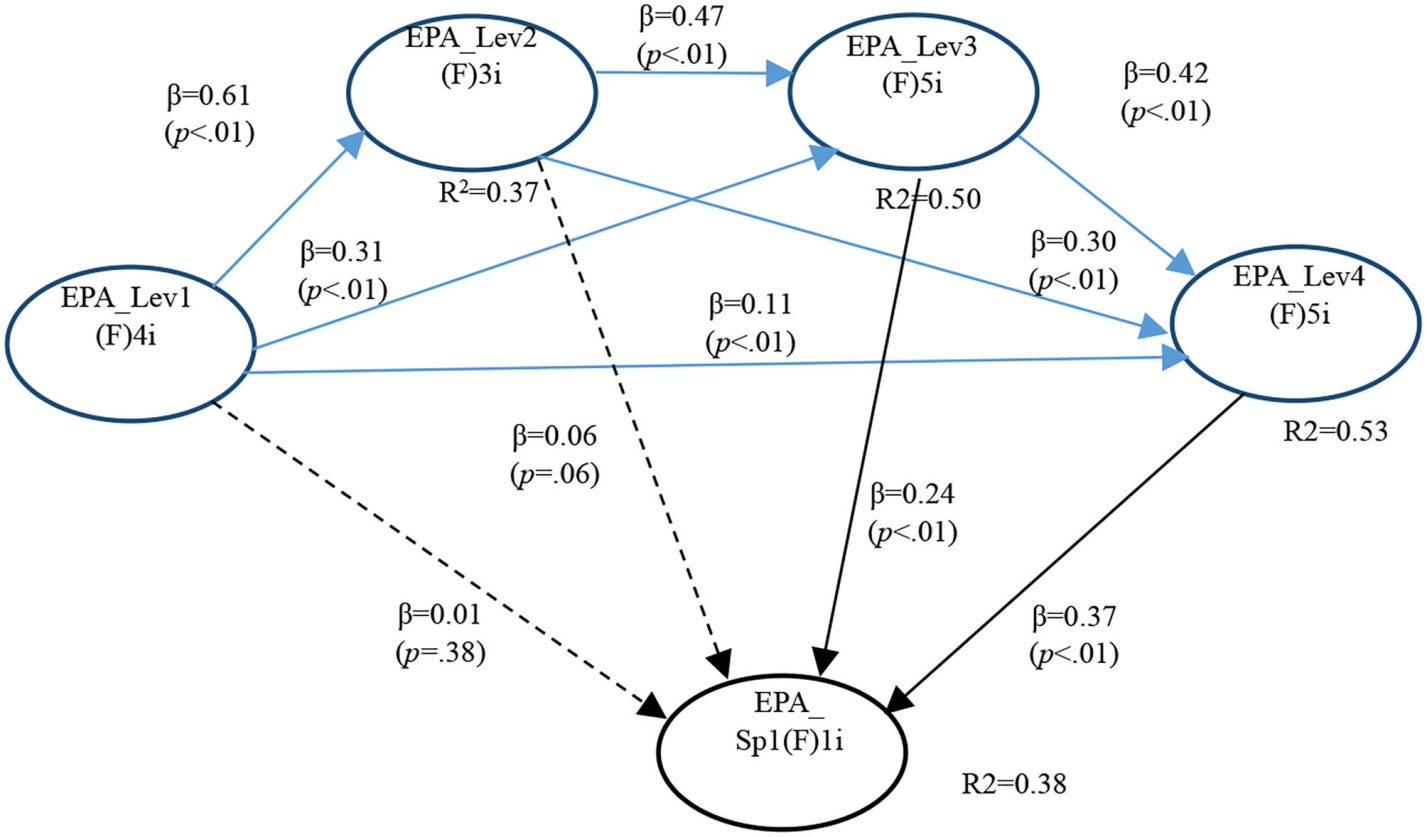
Full model for GI EPA 1.

For analysis purposes, the most informative findings concerned the existence or not of significant relationships between general EPA levels and the SGEPAs under focus. In the case of GI EPA 1, only two significant path Co-efficients were found, one with Level 3 (*P* < 0.001) and the other with Level 4 (*P* < 0.001). This supported both Hypotheses 4a and 5a (Figure 1).

In the second model (General EPA levels and SGEPAs 2), APC = 0.283 (*P* < 0.001), ARS = 0.425 (*P* < 0.001), and AARS = 0.423 (*P* < 0.001), indicating that it matched the requirements of validity and quality.

Multicollinearity was also ruled out (AVIF = 1.945, AFVIF = 2.012). The model fitted well (Tenenhaus GoF = 0.503) without Sympson's paradox problem (SPR = 1.000) or data distortion (SSR = 1.000). Both RSCR and NLBCDR were 1, indicating that the reversal direction had not been improved.

**Figure 2:**
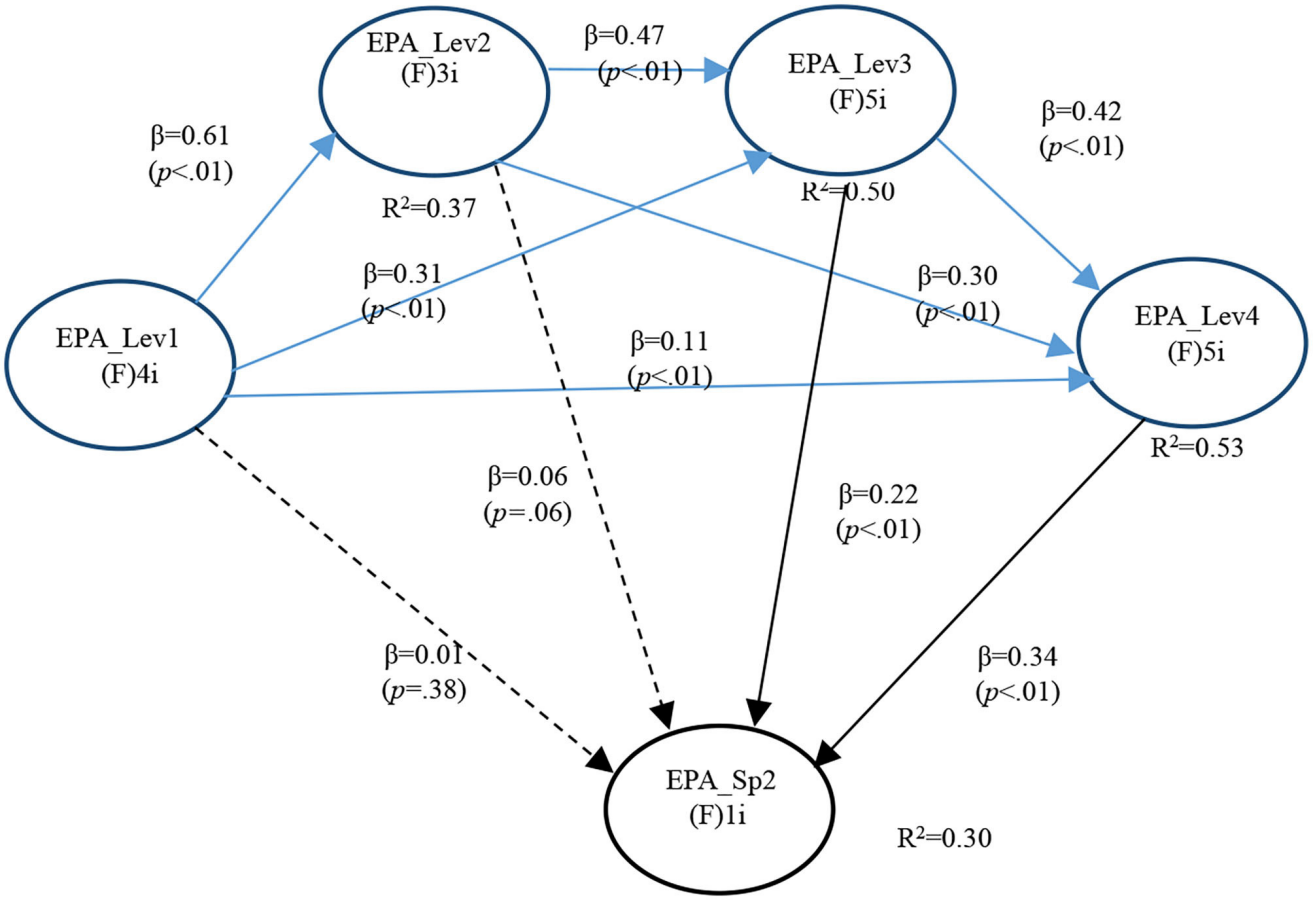
Full model for GI EPA 2.

There were also only two significant path Co-efficients for GI EPA 2. Again, one with Level 3 (*P* < 0.001) and the other with Level 4 (*P* < 0.001). This supported both Hypotheses 4b and 5b (Figure 2 and Table 7).

**Table 7:** Association Co-efficients general EPAs-GI EPA 2 for PLS-SEM.

	**EPA_level 1**	**EPA_level 2**	**EPA_level 3**	**EPA_level 4**
EPA_level 1				
EPA_level 2	0.610^*^			
EPA_level 3	0.313^*^	0.470^*^		
EPA_level 4	0.107	0.295^*^	0.417^*^	
EPA_GI 2	0.013	0.057	0.215^*^	0.338^*^

*P < 0.001.

In the third model (General EPA levels and SGEPAs 3), APC = 0.279 (*P* < 0.001), ARS = 0.415 (*P* < 0.001), and AARS = 0.413 (*P* < 0.001), indicating that it matched the requirements of validity and quality.

Multicollinearity was also ruled out (AVIF = 1.930, AFVIF = 1.998). The model fitted well (Tenenhaus GoF = 0.497) without Sympson's paradox problem (SPR = 0.900). A better value was found for SSR that equals 1. Finally, causality direction is the most suited as seen by both RSCR (0.998) and NLBCDR (1.0) (Table 8).

**Table 8:** Association Co-efficients general EPAs-GI EPA 3 for PLS-SEM.

	**EPA_level 1**	**EPA_level 2**	**EPA_level 3**	**EPA_level 4**
EPA_level 1				
EPA_level 2	0.610^*^			
EPA_level 3	0.313^*^	0.470^*^		
EPA_level 4	0.107	0.295^*^	0.417^*^	
EPA_GI 3	0.038	–0.007	0.260^*^	0.277^*^

*P < 0.001.

**Figure 3:**
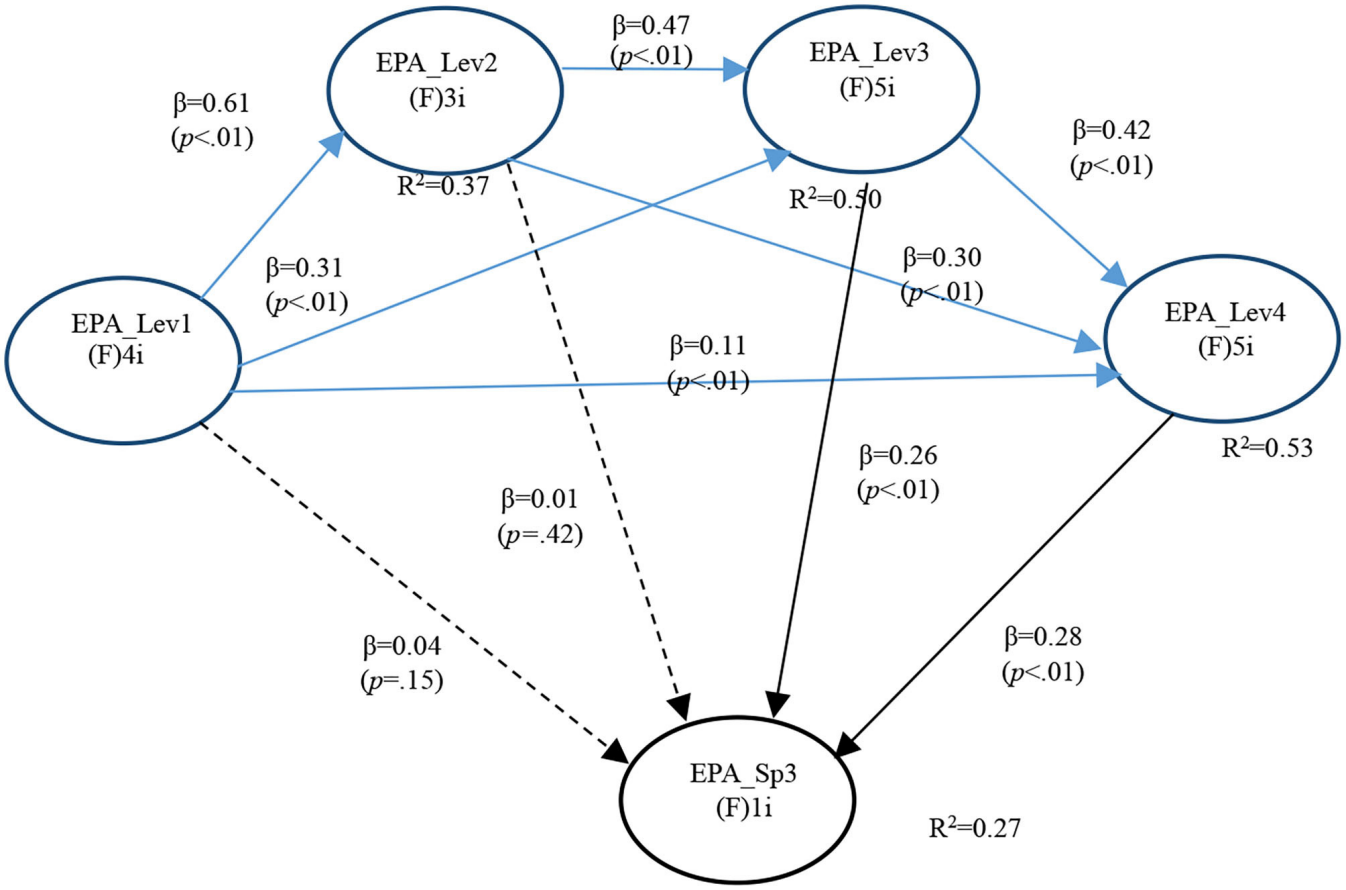
Full model for GI EPA 3.

GI EPA 3 results followed precisely the same pattern of the previous specialized EPAs with the variant that both betas are of similar magnitude. Therefore, one with Level 3 (*P* < 0.001) and the other with Level 4 (*P* < 0.001). This supports both Hypotheses 4c and 5c (Figure 3).

In the fourth model (General EPA levels and SGEPAs 4), APC = 0.283 (*P* < 0.001), ARS = 0.420 (*P* < 0.001), and AARS = 0.418 (*P* < 0.001), indicating that it matched the requirements of validity and quality. Multicollinearity was also ruled out (AVIF = 1.955, AFVIF = 1.982). The model fitted well (Tenenhaus GoF = 0.500) without Sympson's paradox problem (SPR = 1.000) or data distortion (SSR = 1.000). The designed causality direction was also the most suited (RSCR = 1.000 and NLBCDR = 1.000) (Table 9).

**Table 9:** Association Co-efficients general EPAs-GI EPA 4 for PLS-SEM.

	**EPA_level 1**	**EPA_level 2**	**EPA_level 3**	**EPA_level 4**
EPA_level 1				
EPA_level 2	0.610^*^			
EPA_level 3	0.313^*^	0.470^*^		
EPA_level 4	0.107	0.295^*^	0.417^*^	
EPA_GI 4	0.100	0.121^*^	0.186^*^	0.214^*^

*P < 0.001.

**Figure 4:**
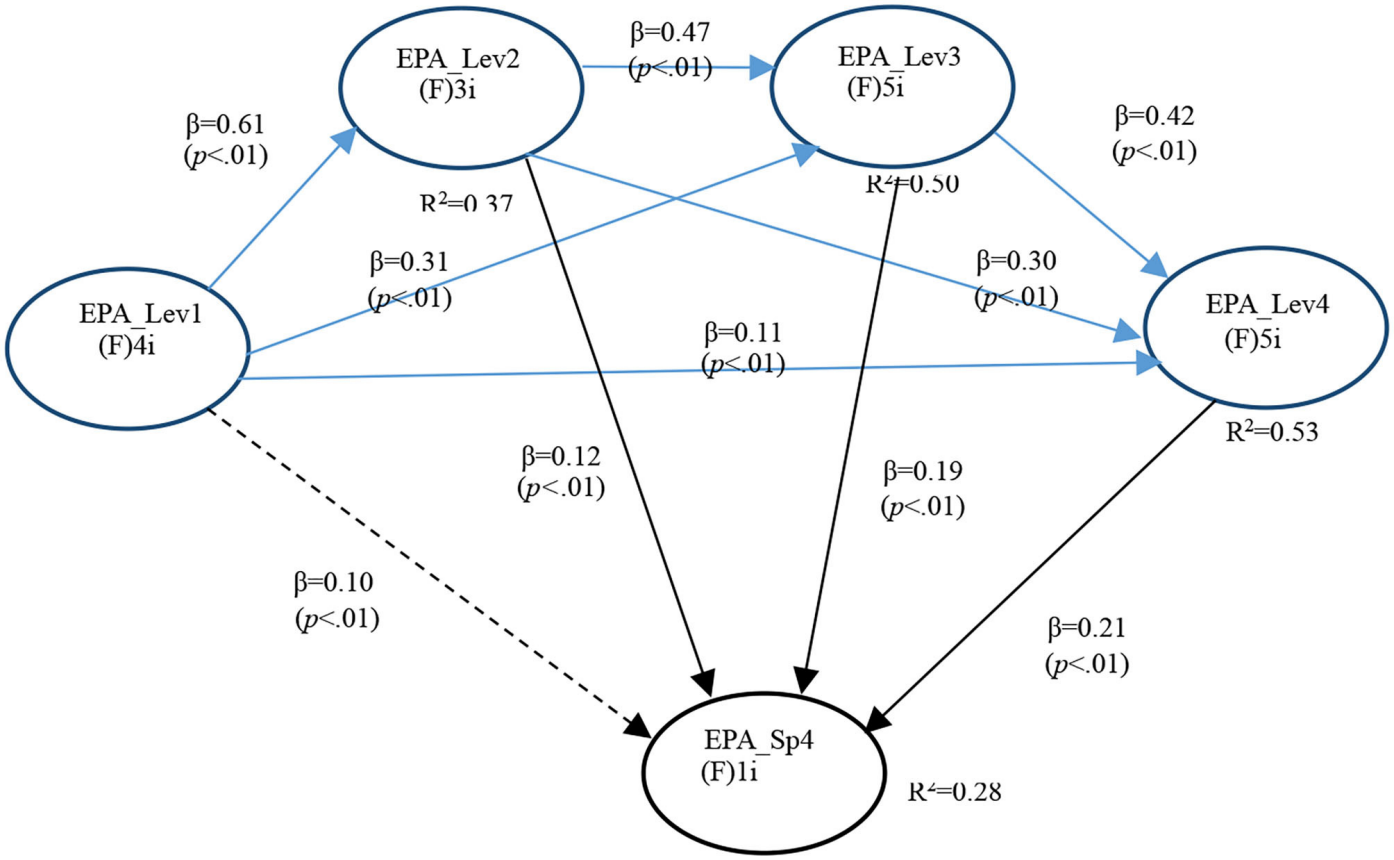
Full model for GI EPA 4.

There are three statistically significant path Co-efficients of modest magnitude for GI EPA 4. Namely with EPA Level 2 (*P* < 0.001), EPA Level 3 (*P* < 0.001), and EPA Level 4 (*P* < 0.001). This supported Hypothesis 4d (Figure 4).

In the last model (General EPA levels and SGEPAs 5), APC = 0.270 (*P* < 0.001), ARS = 0.395(*P* < 0.001), and AARS = 0.393(*P* < 0.001), indicating that it matched the requirements of validity and quality. Multicollinearity was also ruled out (AVIF = 1.897, AFVIF = 1.947). The model fitted well (Tenenhaus GoF = 0.485) without Sympson's paradox problem (SPR = 1.000) or data distortion (SSR = 1.000). The causality direction designed is also the most suited as indicated by both RSCR and NLBCDR achieving a value of 1.0 (Table 10).

**Table 10:** Association Co-efficients general EPAs-GI EPA 5 for PLS-SEM.

	**EPA_level 1**	**EPA_level 2**	**EPA_level 3**	**EPA_level 4**
EPA_level 1				
EPA_level 2	0.610^*^			
EPA_level 3	0.313^*^	0.470^*^		
EPA_level 4	0.107	0.295^*^	0.417^*^	
EPA_GI 5	0.068	0.007	0.133^*^	0.282^*^

*P < 0.001.

**Figure 5:**
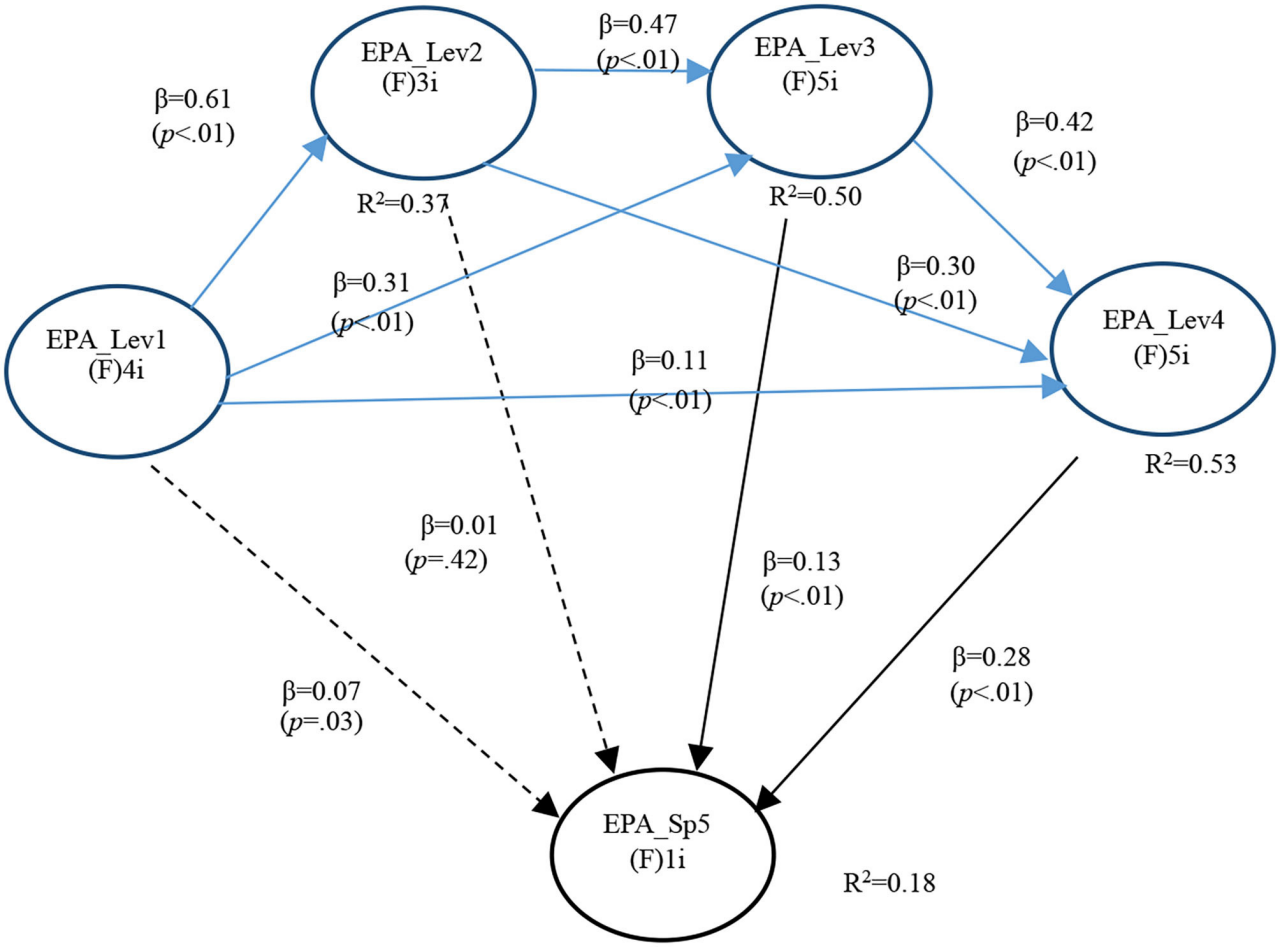
Full model for GI EPA 5.

In the case of GI EPA 5, the predominant pattern is observed with the two more complex general EPA levels showing statistically significant path Co-efficients, namely, with EPA Level 3 (*P* < 0.001) and EPA Level 4 (*P* < 0.001). This supported both Hypotheses 4f and 5f (Figure 5).

Overall, the results of testing prediction models between general and SGEPAs supported the hypothesis. As expected, all statistically significant associations were positive, thus suggesting that specialized EPAs were built on top of general EPAs. Likewise, this significant association was mainly seen at levels 1 and 2. This indicated that levels 1 and 2 were primarily needed to lay the groundwork for levels 3 and 4, and that these higher levels of EPAs were still the most informative for SGEPAs. Given that general EPA levels are designed by consensus as a formative construct, we consider it necessary to explore each general EPA separately to determine which measurement results express homogeneous behavior within each level.

## Discussion

At present, all community hospitals in China are part of the medical consortium project (29). These hospitals act as trainers of general practitioners and health popularizers. Such roles will become mandatory for physicians in these hospitals, and will soon become part of physicians' routine. Therefore, they have to receive relevant training and continue education after graduation to be competent for the new job requirements (30). Before becoming a specialist, one must train as a general practitioner to acquire a full range of medical knowledge (31). Then, combining clinical practice, personal development, and other factors, general practitioners decide whether to pursue further specialist careers. Hospital requirements will in turn become part of the competency model.

To maximize the role of general practitioners in pediatric medical care, optimize and integrate medical resources, and ultimately improve the medical level and patient satisfaction, the EPAs of the AAP and NASPGHAN were used to test whether the MSGM was in line with the practice of pediatrics in China in this study. Medical behavior is the key to measure the competence of doctors. The EPAs theory is closely related to post-competency theory. EPAs include basic practical activities required to train a medical professional, such as pediatric emergency treatment, critical illness identification, and harmonious doctor–patient relationship. The correct implementation of these key clinical practice activities is conducive to improving the work ability and post-competency of doctors.

In China, general pediatricians work in secondary hospitals or community hospitals, and their responsibilities are the diagnosis and treatment of common diseases and child care. Most pediatric gastroenterologists work in tertiary hospitals (general hospitals and children's hospitals), undertake the diagnosis and treatment of pediatric complicated and difficult children with digestive diseases, and are equipped with the operation skills of digestive endoscopy. Therefore, EPAs for general pediatrics and pediatric gastroenterology are different in terms of educational practices, with higher requirements for pediatric gastroenterology doctors than for general pediatricians.

In our study, 776 pediatricians in China were surveyed by questionnaire on EPAs of general pediatricians and pediatric gastroenterology. The qualitative and quantitative statistical analysis, CB model, and PLS-SEM model were conducted on the questionnaire results. The results showed that specialized EPAs depended on general EPAs. A seminar was delivered before the research upon which it was demonstrated that these items reflect the reality of Chinese pediatrics. These EPAs are cleverly broken down into detailed activities according to different medical tasks. Through the quantitative study, we found a positive correlation and complementarity among pediatrician EPAs. EPAs of general pediatricians and digestive pediatricians were also complementary and positively correlated. They also enhanced and reinforced performance indicators. If there is no general practice and skill training of EPAs in the early stage as the basis, the medical ability of pediatric gastroenterology specialty may not be achieved in the later stage. This study also found that “professionalism” and “medical knowledge” were significantly positively correlated with EPAs of general pediatricians and pediatric gastroenterology, suggesting that these two competencies are the basis of other competencies.

This research focused on the approaches and methods of post-graduate medical education and continuing education and studied the growth patterns of digestive pediatricians, a professional group that is in short supply in China's medical industry (7). The study confirmed that the number of pediatricians is declining significantly due to a variety of reasons, including government policies, hospital management, department performance, compensation, and education patterns (32, 33). This study attempts to establish MSGM for community hospitals based on EPAs, which is helpful to relieve the pressure of pediatricians' loss through this new and effective training method in China's medical reform.

This thesis is a prospective study of the digestive pediatrician education system, an exploration of the digestive endoscopy pediatrician panel of the National Health Commission of the People's Republic of China. MSGM serves as a way toward the improvement and exploration of the education system for pediatricians after graduation and for continuing education. Meanwhile, it also helps lay the foundation for a clinical pediatrician's career after graduation and for continuing education.

Entrustable professional activities have a close relationship with the actual clinical and teaching environment and national conditions. Although the MSGM established according to EPAs has certain theoretical significance and reference value for the improvement and exploration of post-graduation and continuing education system of Pediatricians in China, in view of the differences in a clinical teaching environment and national conditions between China and the United States, it is imperative to establish a Chinese EPAs suitable for pediatricians. In future studies, it is necessary for scholars to explore China's own EPAs based on unique national conditions.

## Data Availability Statement

The original contributions presented in the study are included in the article/Supplementary Material, further inquiries can be directed to the corresponding author/s.

## Ethics Statement

Ethics review and approval/written informed consent was not required as per local legislation and institutional requirements.

## Author Contributions

NCR directed the entire research process. VT and RZ designed the study. SG and NL drafted the manuscript. XW carried out the data collection. YY participated in the data processing. All authors reviewed and approved the final version of the manuscript.

## Funding

This research was supported by the grants from the Management research project of Shanghai Shenkang Hospital Development Center (Grant No. 2021SKMR-18).

## Conflict of Interest

The authors declare that the research was conducted in the absence of any commercial or financial relationships that could be construed as a potential conflict of interest.

## Publisher's Note

All claims expressed in this article are solely those of the authors and do not necessarily represent those of their affiliated organizations, or those of the publisher, the editors and the reviewers. Any product that may be evaluated in this article, or claim that may be made by its manufacturer, is not guaranteed or endorsed by the publisher.

## Abbreviations

WHO: World Health Organization
EPAs: entrustable professional activities
AAP: American Academy of Pediatrics
KMO: Kaiser–Meyer–Olkin
MSGM: medical service-groups model
APC: average path Co-efficient
ARS: average R-squared
AARS: average adjusted R-squared
AVIF: average block VIF
AFVIF: Average full collinearity VIF
GoF: Tenenhaus GoF
SPR: sympson's paradox ratio
RSCR: R-squared contribution ratio
SSR: software incorporates an index
NLBCDR: non-linear bivariate causality direction ratio
CR: composite reliability
Lev: level
SGEPAs: specialized gastrointestinal EPAs
CITC: corrected item total correlation.
