# Supplementary material for: An Assessment Framework for the Training of General Practitioners and Specialists Based on EPAs
Source: Front Public Health. 2022 Jul 7;10:896097. doi: 10.3389/fpubh.2022.896097 (PMC9300895; doi:10.3389/fpubh.2022.896097)
Supplement: Supplementary file 1 [file Data_Sheet_1.pdf]

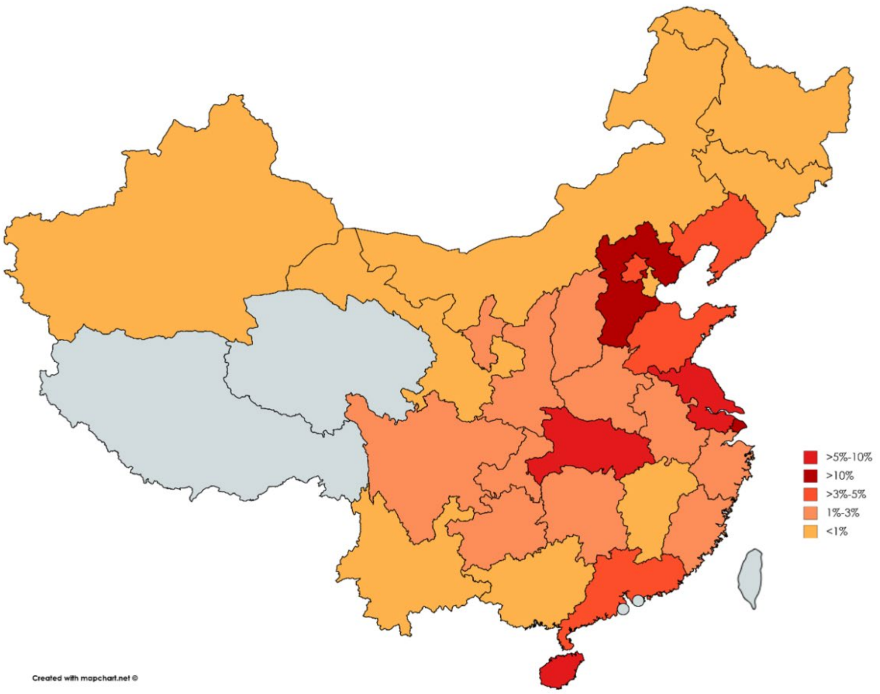

Supplement figure 1 Sample geographic map

Supplement table 1 Sample geographic origin

Province	N	%	Province	N	%
Anhui	17	2.2%	Jiangsu	43	5.5%
Beijing	24	3.1%	Jiangxi	7	0.9%
Chongqing	21	2.7%	Jilin	6	0.8%
Fujian	9	1.2%	Liaoning	37	4.8%
Guangdong	28	3.6%	Ningxia	13	1.7%
Guangxi	3	0.4%	Overseas	11	1.4%
Gansu	1	0.1%	Shan´xi	9	1.2%
Guizhou	9	1.2%	Shandong	29	3.7%
Hainan	39	5.0%	Shanghai	148	19.1%
Hebei	157	20.3%	Shanxi	11	1.4%
Heilongjiang	4	0.5%	Sichuan	17	2.2%
Henan	10	1.3%	Tianjin	2	0.3%
Hubei	75	9.7%	Xinjiang	2	0.3%
Hunan	12	1.5%	Yunnan	6	0.8%
Inner Mongolia	5	0.6%	Zhejiang	20	2.6%

Supplement table 2 Type of pediatrician per hospital grade

X\Y	Teaching Hospitals	Central Hospitals	Community Hospitals	Subtotal
Pediatrician (General \Childcare)	162 (57.4%)	107 (37.9%)	13 (4.6%)	282
Pediatricians (Specialists)	294 (81.9%)	62 (17.3%)	3 (0.8 %)	359
General Physicians in Communities	1 (8.3%)	6 (50%)	5 (41.7%)	12

Supplement table 3 Type of pediatrician per nature of hospital

X\Y	Special Hospitals	General Hospitals	Community Hospitals	Subtotal
Pediatrician (General \Childcare)	73 (25.9%)	195 (69.1%)	14 (5.0%)	282
Pediatricians (Specialists)	209 (58.2%)	148 (41.2%)	2 (0.6%)	359
General Physicians in Communities	2 (16.7%)	2 (16.7%)	8 (66.7%)	12

Supplement table 4 Commonalities of full 17 general EPAs factor analysis

EPAs	Extraction
B1 Establishing and maintaining working relationships	.350*
B2 Using the screening tools and conducting related interpretation	.546
B3 Carrying physical examination	.476*
B4 Handle common pediatric emergencies	.523
B5 Establishing a harmonious relationship with patients and their family members	.433*
B6 To have an understanding of basic community services	.419*
B7 Identifying patients who require surgery.	.530
B8 Transition between pediatricians and adult medical corresponding departments	.417*
B9 Identifying and treating common behavior and mental health problems	.365*
B10 Determine and prioritize treatment in order of importance and urgency.	.554
B11 Focus on learning purposes, in a timely manner to retrieve relevant information	.482*
B12 Use referral guidelines to make decisions.	.532
B13 provide patients with a cost-effective diagnosis and treatment plan.	.631
B14 Using data to identify high-risk populations, epidemiological principles.	.570
B15 Act as a management leader in medical care	.488*
B16 Transfer from clinic to inpatient and vice versa.	.566
B17 Have the ability to perform routine diagnostic and treatment operations	.328*

* signals cases of insufficient commonality

Supplement table 5 Rotated matrix for general EPAs

	Factor	
	Systemic	Priorizing
B13 provide patients with a cost-effective diagnosis and treatment plan.	.822	.079
B14 Using data to identify high-risk populations, epidemiological principles.	.770	.175
B12 Use referral guidelines to make decisions.	.721	.255
B4 Handle common pediatric emergencies	.158	.764
B7 Identifying patients who require surgery	.142	.751
B10 Determine and prioritize treatment in order of importance and urgency.	.173	.739

Extraction: Principal components. Rotation: Varimax with Kaiser normalization.

a. Rotation converged in 3 iteractions.

Supplement table 6 Commonalities of full 5 SGEPAs factor analysis

SGEPAs	Extraction
B1 Establishing and maintaining working relationships	.600*
B2 Using the screening tools and conducting related interpretation	.628
B3 Carrying physical examination	.597*
B4 Handle common pediatric emergencies	.501
B5 Establishing a harmonious relationship with patients and their family members	.477*

Supplement table 7 general and specialized gastrointestinal EPAs reliability

General EPAs	CITC	Item Deleted α Coefficient	Cronbach α Coefficient	SGEPAs	CITC	Item Deleted α Coefficient	Cronbach α Coefficient
B1	.399	.881	.883	B18	.612	.754	.801
B2	.469	.879		B19	.634	.747	
B3	.496	.877		B20	.616	.754	
B4	.511	.877		B21	.543	.775	
B5	.575	.874		B22	.525	.784	
B6	.548	.875					
B7	.543	.876					
B8	.535	.876					
B9	.521	.876					
B10	.482	.878					
B11	.540	.876					
B12	.563	.875					
B13	.501	.877					
B14	.578	.874					
B15	.492	.878					
B16	.615	.873					
B17	.479	.878					

Supplement table 8 Interrater consensus

Level	EPAs	Rationale	Y/N
1	B2 Using the screening tools and conducting related interpretation<br>B3 Carrying physical examination<br>B1 Establishing and maintaining working relationships<br>B5 Establishing a harmonious relationship with patients and their family members	Establishing a harmonious relationship with patients and their family members". When faced with any clinical situation, a pediatrician is required to correctly use screening tools to gather information. This information will be more accurate when the physician is capable of establishing a positive relationship with the patient's family as well as colleagues. A family that is trusting and willing to cooperate will disclose more information. Likewise, colleagues with whom one can have an open communication channel will also contribute to triangulate and clarify doubtful situations.	
2	B4 Handle common pediatric emergencies<br>B6 To understand basic community services<br>B14 Using data to identify high-risk populations, epidemiological principles	When facing emergencies, a pediatrician must be capable of considering macro level information that relates with risk groups, epidemiology as well as knowing well the services that usually refer emergency cases to pediatricians, i.e. community centers	
3	B10 Determine and prioritize treatment in order of importance and urgency<br>B8 Transition between pediatricians and adult medical corresponding departments<br>B16 Transfer from clinic to inpatient and vice versa<br>B7 Identifying patients who require surgery<br>B9 Identifying and treating common behavior and mental health problems	Many cases will require the intervention of other medical area professionals. These cases require a sense of importance and know how to transfer to other services to provide for clinical needs that may fall outside the scope of pediatricians.	
4	B12 Use referral guidelines to make decisions<br>B11 Focus on learning purposes, in a timely manner to retrieve relevant information<br>B13 Provide patients with a cost-effective diagnosis and treatment plan<br>B15 Act as a management leader in medical care<br>B17 Be able to perform routine diagnostic and treatment operations	Delivering best practice service implies knowing and observing referral guidelines while keeping a focus not only on treatment but on the need to lead oneself into learning more while leading others. This level is the most complex as it comprehends practices that are usually allocated only to pediatricians in community hospitals	

Supplement table 9 Direct and indirect effects in CB models

IV	Med1	Med2	DV	Direct effect	Indirect effect (unstandardized)	95%CI LB UB	Hypothesis
Lev1	Lev2			.6593		[.5988; .7198]	
Lev1		Lev3		.3017		[.2400; .3635]	
	Lev2	Lev3		.4238		[.3667; .4809]	
Lev1			Lev4	.1025		[.0500; .1550]	
	Lev2		Lev4	.1802		[.1284; .2320]	
		Lev3	Lev4	.3427		[.2860; .3995]	
Lev1	Lev2	Lev3			.1188	[.0796; .1621]	1
Lev1		Lev3	Lev4		.1034	[.0746; .1340]	2
Lev1	Lev2	Lev3	Lev4		.0958	[.0742; .1193]	3

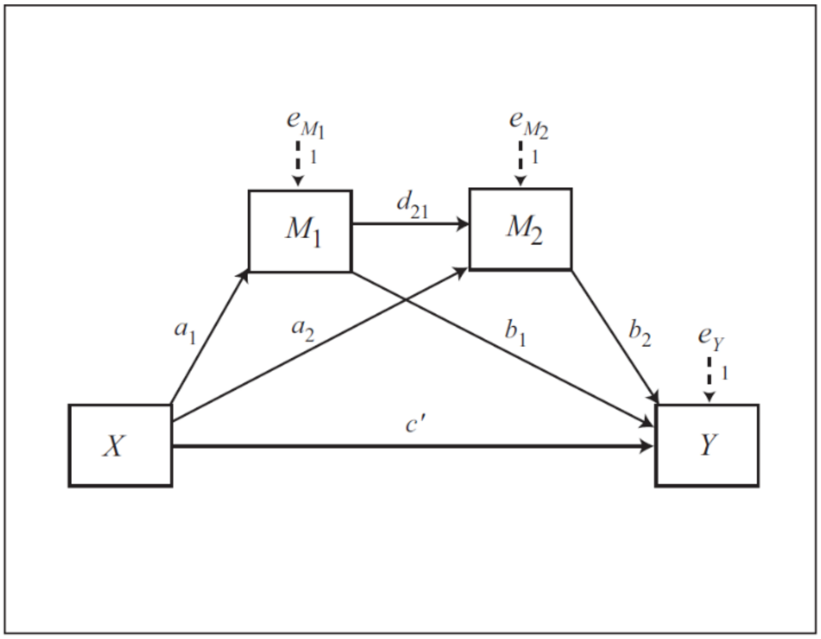

Supplement figure 2 Statistical model of sequential mediation

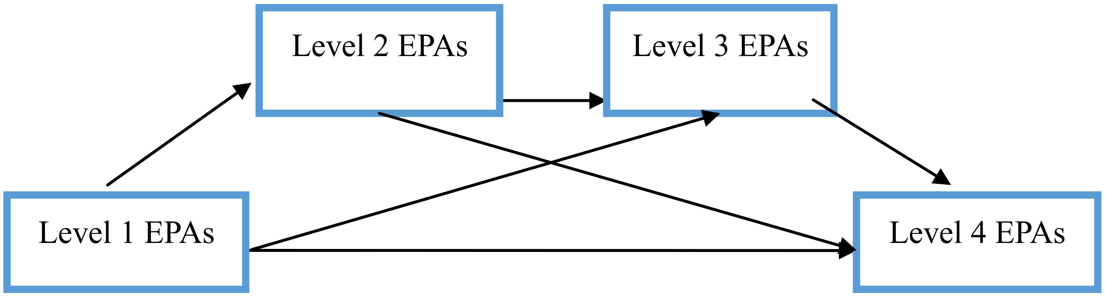

Supplement figure 3 The schematic of MSGM

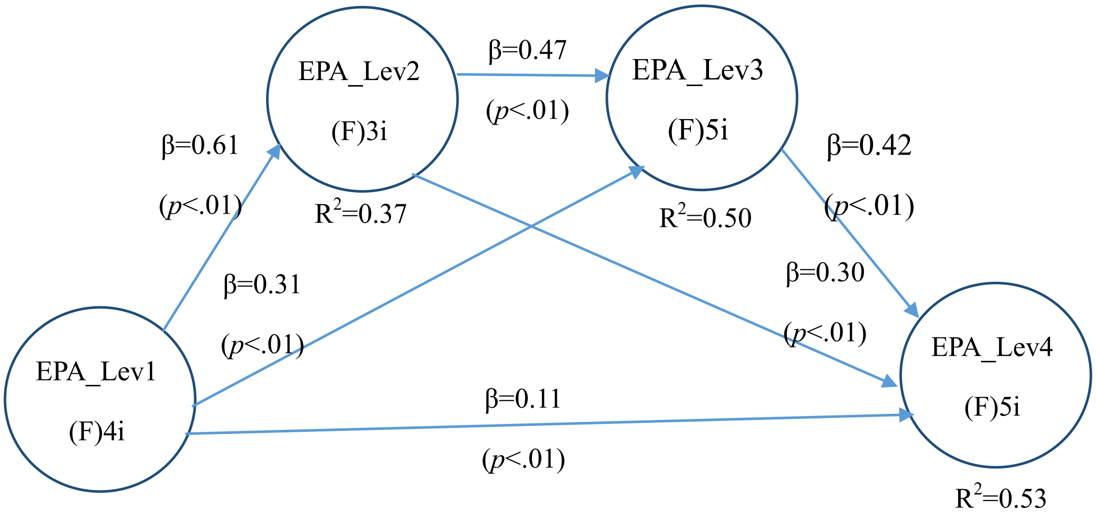

Supplement figure 4 Full model path coefficients

Supplement table 10 Direct and indirect effects in PLS-SEM

IV	Med1	Med2	DV	Direct effect	Indirect effect (unstandardized)
Lev1	Lev2			.610**	
Lev1		Lev3		.313**	
	Lev2	Lev3		.470**	
Lev1			Lev4	.107**	
	Lev2		Lev4	.295**	
		Lev3	Lev4	.417**	
Lev1	Lev2	Lev3			.287**
Lev1		Lev3	Lev4		.310**
Lev1	Lev2	Lev3	Lev4		.120**

** *P* <.01
